# Weighted Gene Co-expression Network Analysis of the Dioscin Rich Medicinal Plant *Dioscorea nipponica*

**DOI:** 10.3389/fpls.2017.00789

**Published:** 2017-06-07

**Authors:** Wei Sun, Bo Wang, Jun Yang, Weihao Wang, An Liu, Liang Leng, Li Xiang, Chi Song, Shilin Chen

**Affiliations:** ^1^Key Laboratory of Beijing for Identification and Safety Evaluation of Chinese Medicine, Institute of Chinese Materia Medica, China Academy of Chinese Medical SciencesBeijing, China; ^2^Guangdong Provincial Key Laboratory of Applied Botany, South China Botanical Garden, Chinese Academy of SciencesGuangzhou, China; ^3^Hubei Institute for Food and Drug ControlWuhan, China

**Keywords:** *Dioscorea nipponica*, dioscin, RNA-seq, WGCNA

## Abstract

Dioscorea contains critically important species which can be used as staple foods or sources of bioactive substances, including *Dioscorea nipponica*, which has been used to develop highly successful drugs to treat cardiovascular disease. Its major active ingredients are thought to be sterol compounds such as diosgenin, which has been called “medicinal gold” because of its valuable properties. However, reliance on naturally growing plants as a production system limits the potential use of *D. nipponica*, raising interest in engineering metabolic pathways to enhance the production of secondary metabolites. However, the biosynthetic pathway of diosgenin is still poorly understood, and *D. nipponica* is poorly characterized at a molecular level, hindering in-depth investigation. In the present work, the RNAs from five organs and seven methyl jasmonate treated *D. nipponica* rhizomes were sequenced using the Illumina high-throughput sequencing platform, yielding 52 gigabases of data, which were pooled and assembled into a reference transcriptome. Four hundred and eighty two genes were found to be highly expressed in the rhizomes, and these genes are mainly involved in stress response and transcriptional regulation. Based on their expression patterns, 36 genes were selected for further investigation as candidate genes involved in dioscin biosynthesis. Constructing co-expression networks based on significant changes in gene expression revealed 15 gene modules. Of these, four modules with properties correlating to dioscin regulation and biosynthesis, consisting of 4,665 genes in total, were selected for further functional investigation. These results improve our understanding of dioscin biosynthesis in this important medicinal plant and will help guide more intensive investigations.

## Introduction

*Dioscorea* (true yams) is a large monocot genus of about 450 species of herbaceous vines that are dioecious and rhizomatous or tuberous. These species have attracted a great deal of attention since they appear similar to dicots (Mignouna et al., 2009) and some are critically important species with edible tubers which serve as a major staple food (Tang et al., 2013) or as a source of bioactive substances used in a range of applications related to human health (Govaerts et al., 2007; Sautour et al., 2007; Kroymann, 2011).

For example, *D. nipponica* has been developed into patent medicine, which is effective in the prevention and treatment of cardiovascular disease and are sold in many countries.

*Dioscorea nipponica*, a rhizomatous Dioscorea plant, is the most widely distributed and the resource-richest species of Dioscorea in China. Its dried rhizome is used in Chinese medicine to treat symptoms such as rheumatoid arthritis, traumatic injuries, bronchitis, coronary heart disease and rheumatic fever (Yang et al., 2006; Sautour et al., 2007). In addition, recent studies have shown that its extracts exhibit a curative effect toward climacteric syndrome and obesity (Kwon et al., 2003; Dewick, 2011), as well as showing inhibitory effects on many cancers, including melanotic tumors, oral cavity carcinomas and neuroblastomas (Ho et al., 2011; Chien et al., 2012; Rahman et al., 2014). Sterols and their derivatives are suggested to be the major active ingredients in *D. nipponica* rhizomes. In particular, diosgenin possesses a variety of bioactivities, such as anti-inflammatory, anti-hyperlipidemia, and anti-aging (Huo et al., 2004; Tada et al., 2009; Yan et al., 2009; Gong et al., 2010), and can make up as much as 2.6% of the dry weight of the rhizome (Sautour et al., 2007). Diosgenin is the most important starting material for the synthesis of more than 400 kinds of steroid hormone drugs, such as progesterone, oxytocin, prednisone acetate, budesonide, and oral contraceptives, which can’t been synthesized from scratch due to their complex molecular structure. Given its pharmacological properties and high diosgenin content, *D. nipponica* has great potential for development and medicinal applications. However, reliance solely on naturally growing plants as a production system has led to *D. nipponica* being listed as an endangered species.

In order to address this shortage, efforts have been made to increase diosgenin yield, such as improving the extraction rate of diosgenin (Yoon and Kim, 2008) or treating plant with inducers to up-regulate the diosgenin biosynthesis pathway (De and De, 2005; Diarra et al., 2013), although engineering the metabolic pathways to enhance the biosynthesis of the target product is probably a more effective approach (Bauer et al., 2011; Gu et al., 2012; Bartley et al., 2013). Previous studies have shown that introduction and overexpression the gene encoding the rate-limiting enzyme sterol-C_24_-methyltransferase type 1 can effectively improve the yield of sterols in *Nicotiana tabacum* (Holmberg et al., 2002, 2003), and technological advances now allow a better understanding of plant systems and will increase the practical potential of metabolic engineering (Oksman-Caldentey and Saito, 2005; Häkkinen et al., 2013). However, fundamental knowledge about the metabolic networks and genome of Dioscorea is utterly lacking, which has hindered in-depth investigation and application of this plant (Wang et al., 2015). High-throughput sequencing and systematic analysis of the genes involved in dioscin biosynthesis in this species are essential groundwork for future investigations.

The objectives of this study are to understand the genetic basis of the biosynthesis and metabolic regulation of dioscin in *D. nipponica*. To do so, a high throughput sequencing strategy and weighted gene co-expression network analysis (WGCNA) were employed to investigate sterol metabolism in *D. nipponica*, based on the idea that genes which are co-expressed under multiple conditions tend to be functionally related (Stuart et al., 2003; Ihmels et al., 2004; Aoki et al., 2007). Arabidopsis brassinosteroid level, a kind of another sterol compound, can be further increased by treatment with methyl jasmonate (MeJA) (Kim et al., 2013). The expression patterns and co-expression networks of transcripts in different organs and following MeJA treatments were therefore analyzed to identify and understand dioscin-related genes. The results presented here firstly contribute to our understanding of the synthesis and regulation of complex sterols in *D. nipponica*, providing the foundations for further analysis of this pathway and the potential manipulation of this pharmaceutical resource in this important medicinal species.

## Materials and Methods

### Plant Materials

*Dioscorea nipponica* plants were collected from the medicinal botanical garden of the Nanyang Institute of Technology in Henan province, China. Young healthy rhizomes were then cut into an equal number of segments of 3–5 cm and planted in a field nursery at the Institute of Chinese Materia Medica in the China Academy of Chinese Medicinal Sciences. Flowers, leaves, stems, bubs and rhizomes were collected at the same growth period and stored in liquid nitrogen until use.

### MeJA Treatment

For the MeJA treatment, healthy *D. nipponica* plants were carefully collected and transplanted into a soilless culture system with quartz sand (20–40 mesh) used as a supporting medium. All of the plants were cultured in the same conditions (25°C, humidity 60%, light/dark 16/8 h) with the same volume of sugar-free 1/2 Murashige and Skoog medium in an artificial climate incubator for 30 days, after which the culture medium was replaced with a medium that contained MeJA (Sigma–Aldrich, America) at a final concentration of 200 μM; samples were collected at 0, 12, 24, 36, 48, 120, and 240 h after treatment. Three biological replicates were done for each treatment.

### Chemical Content Analysis

Rhizome samples collected at each period were ground into powder, and 0.1 g of the powder was accurately weighted and soaked overnight in 5 mL chloroform. The solution was then filtered and treated with ultrasound for 60 min, vaporizing the chloroform. The residues were dissolved in 200 μL methanol-chloroform (1:1) and then centrifuged at 16,000 rpm for 5 min to prepare the samples for LC-MS/MS analysis. Two technical replicates were performed for each of the three biological samples. An Agilent XBD C18 column (1.8 μm, 4.6 × 50 mm) was used as the chromatographic column; the flow rate was 0.5 mL/min and the column temperature was set at 35°CC. A solution of 50% methanol, 25% acetonitrile, 25% isopropyl alcohol and 0.025% methanoic acid was used as the mobile phase. For the tandem mass spectrometry analysis, positive ion atmospheric pressure chemical ionization was selected with the following settings: atomization gas, 30 psi; atomization temperature, 350°C; nitrogen flow rate, 5 L/min; corona discharge needle, 4 μA; capillary voltage, 3,500 V. The standard curve of the analysis can be found in **Supplementary Figure S1**.

### RNA Extraction, and Sequencing

Total RNAs were extracted from the untreated and MeJA-treated samples using an RNAprep Pure Plant Kit according to the manufacturer’s instructions (Tiangen Biotech, China). The integrity and quantity of the RNA samples were analyzed by 1.2% agarose gel electrophoresis and with a NanoDrop 2000C Spectrophotometer (Thermo Scientific, USA), Qubit^®^2.0 Fluorometer (Invitrogen, USA) and Agilent 2100 Bioanalyzer (Agilent, USA). The RNA sequencing libraries were constructed with an RNA Library Prep Kit for Illumina^®^(NEB #E7420S/L) according to the manufacturer’s instructions, and the sequencing was carried out on an Illumina HiSeq2000 high-throughput sequencer. The raw reads were filtered using the NGS QC Toolkit (Patel and Jain, 2012) to remove reads with more than 30% low quality bases (Q < 30) and reads with more than 10% non-ATCG (N); low quality regions of the reads were trimmed using trimmingReads.pl.

### Data Analysis

All the clean data were integrated into a pool for transcriptome assembly. The Trinity software (Grabherr et al., 2011) was employed to assemble the transcripts and analyze differential gene expression in different organs and in the MeJA-treated samples from different periods. Gene expression levels were calculated and normalized to reads per kilobase of exon model per million mapped reads (RPKM) (Mortazavi et al., 2008). A cutoff of RPKM > 1.0 was used to detect expressed genes. Heatmaps were generated using the R package heatmap.3.R. The genes with four times of gene expression level from different periods induced by MeJA treatment than that of control individuals were regarded highly expressed genes. Annotation of the transcripts was performed with the BLASTX program against the Nr database and the swissport/uniport database with threshold of an *E*-value less than 1E^-6^. TransDecoder^1^ and InterProScan5 (Jones et al., 2014) were used to identify candidate coding regions (CDS) and classify the protein functions of the assembled transcripts, respectively. Gene Ontology (GO) analysis was performed using the blast2go program (Conesa et al., 2005), and GO annotation graphs were plotted using the online tool Web Gene Ontology Annotation Plot (WEGO^2^). GO enrichment analysis was carried out with run_GOseq.pl, a script in the Trinity software. Expression data were normalized by square root transformation and used to infer co-expression gene network modules with the WGCNA R software package (Zhang and Horvath, 2005; Langfelder and Horvath, 2008) using step-by-step network construction and the module detection method; a proper power-law coefficient β was selected using the soft-thresholding method, and a dynamic hierarchical tree cut algorithm was used to detect the co-expression modules. Responsive transcriptional factors induced by MeJA were annotated using PlantTFDB 4.0^3^.

## Results and Discussion

### Global Analysis of the Genes in *D. nipponica*

The transcriptome sequences were assembled using Trinity from a 52 gigabase pool of 125 bp paired-end reads generated from more than 4.3 gigabases of data from each of the 12 samples. All the transcriptome data (accession: SRP104221) were deposited in NCBI database. The GC content of the sequences was 43.70%, and a total of 153,924 genes with an N50 contig of 1,114 bp and 210,612 transcripts with an N50 contig of 913 bp were obtained. The quality of the assembled sequences was evaluated by examining the distribution of the percent of a protein’s length covered for the top matching database entries; there were 6,182 proteins that represented by nearly full-length transcripts with more than 90% alignment coverage and 8,626 proteins that each matched a Trinity transcript for more than 80% of their protein length (**Supplementary Table S1**), indicating that the quality of the sequences is sufficient for use as reference data for further analyses (Haas et al., 2013). Sequence annotation was performed against the Nr and uniport/swissport database using the BLAST algorithm. 120,798 genes, accounting for 78.5% of the total, were successfully annotated; these genes were associated with 73,189 GO terms, delineating the known molecular functions, biological processes, and cellular components for particular genes (Ashburner et al., 2000). These results provide a general overview of the assembled transcriptome which was used for further analysis of gene expression.

**FIGURE 1:**
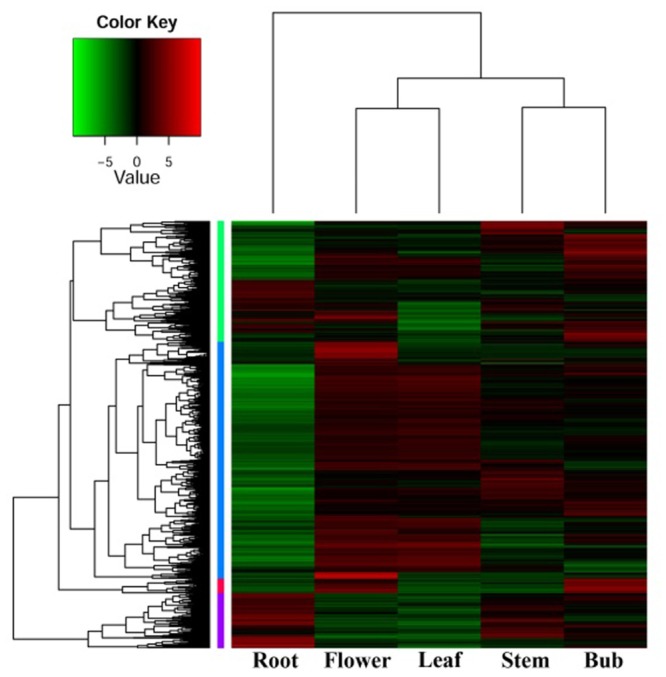
**Biosynthetic pathways of dioscin derived from cholesterol and plant C-24 alkylsterols.** Plant C-24 alkylsterols and cholesterol, the common intermediates in dioscin biosynthesis, are biosynthesized from cycloartenol.

In order to investigate organ-specific expression of genes in *D. nipponica*, a hierarchical agglomerative clustering method was employed to analyze differential gene expression and identify genes with an expression change of more than four times with a significance level of *p* ≤ 1e^-4^. Significant differences in gene expression were detected between the organs investigated. Expression patterns of genes from the underground portions (rhizomes) and aerial portions (flowers, leaves, stems, and buds) clustered into different branches, with the expression pattern in flowers being closer to that of leaves and the expression pattern in stems clustering together with buds in a sub-branch (**Figure 1**). This is consistent with the idea that genes with similar functions will have similar expression patterns; for example, genes involved in some metabolic pathways were expressed in both flowers and leaves because these pathways are needed in both organs (Wang et al., 2015). Furthermore, the genes also clustered into four groups on the hierarchical agglomerative clustering diagram, marked on the left of the heatmap with green, blue, red, and purple bars (**Figure 1**). The green group contains 1,050 genes; the blue group contains 2,064 genes; the red group contains 125 genes; and the purple group contains 482 genes. The heatmap shows that the genes in the blue group were highly expressed in flowers and leaves, while genes in the purple group showed high expression levels in stems and especially in rhizomes.

Gene ontology enrichment analysis was used to functionally characterize the genes in these groups. Genes in the green group matched 95 GO terms; blue genes were annotated with 172 GO terms; purple genes grouped into 62 terms; and only 16 terms were retrieved for genes from the red group. The genes in the green group were mainly related to oxidation-reduction reactions, cell membrane component biosynthesis, and sugar and lipid metabolism. Genes in the blue group mainly involved in photosynthesis and carbohydrate metabolism, which is consistent with their high expression in leaves. Genes marked by the red bar mainly take part in the physiological processes of hydrolysis reactions and signal transduction. Genes in the purple group were mainly involved in transcriptional regulation and stress response involving the production of chemical defense components, such as sterols (Kohara et al., 2005; Chaturvedi et al., 2012), which are part of the stress response in *D. nipponica*; this is consistent with their high expression in the rhizomes, which are the tissue richest in dioscin.

### Identification of the Genes Related to Dioscin Biosynthesis

**FIGURE 2:**
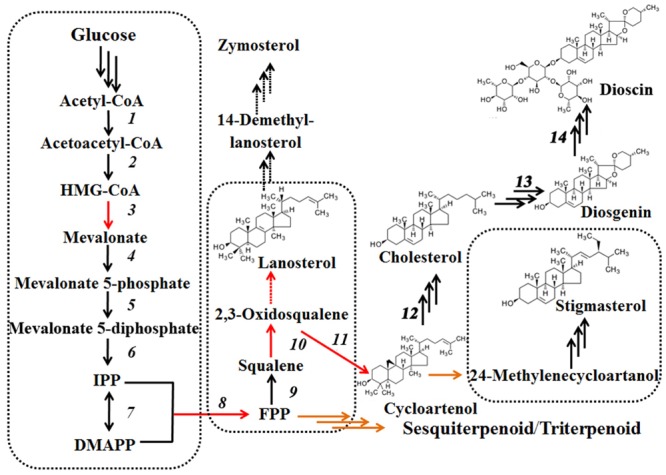
**A differential expression heatmap showing the expression of genes from different *D. nipponica* tissues**.

**FIGURE 3:**
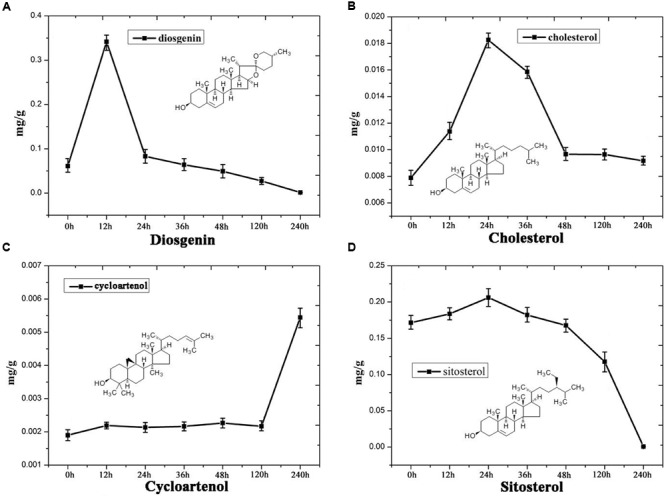
**Content analysis showing the levels of (A)** diosgenin, **(B)** cholesterol, **(C)** cycloartenol and **(D)** sitosterol in MeJA-treated *D. nipponica* roots.

**FIGURE 4:**
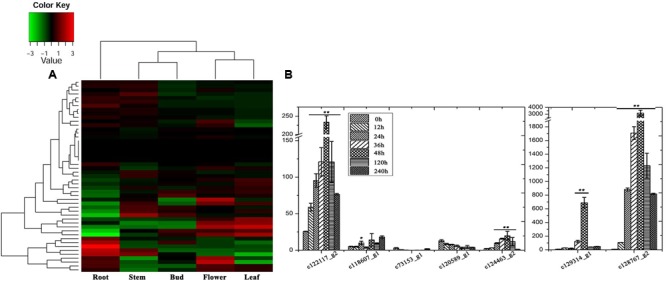
**Expression analysis of the glycosyltransferase genes. (A)** Organ-specific expression analysis of the glycosyltransferase genes in *D. nipponica*. **(B)** Expression analysis of the glycosyltransferase genes highly expressed in roots in of MeJA-treated organs. Error bars are SD. Statistical significance was determined by Student’s *t*-test (^∗^*p* ≤ 0.01; ^∗∗^*p* ≤ 0.001).

Steroidal saponins are widely distributed in the plant kingdom, and more than 10,000 kinds of steroidal saponin compounds have been discovered, including spimstanol saponin, fumstanol saponin, and cholestenol saponin. The biosynthetic pathways of cholestenol saponins, such as the important phytohormones sitosterol and brassinosteroids (Symons and Reid, 2004), have been intensively studied (Chu et al., 2005), while the biosynthesis of diosgenin, a spimstanol saponin, remains poorly understood. Previous work using isotope labeling has suggested that spimstanol saponins are derived from cholesterol (Bennett and Heftmann, 1965; Tomita and Uomori, 1971), and reports have increasingly shown that cholesterol is the precursor of the C24-desmethyl sterols (McCue et al., 2005, 2006, 2007; Itkin et al., 2011, 2013; Petersson et al., 2013), providing further evidence to support the notion that dioscin is derived from cholesterol (**Figure 2**). C24 methylation is the main divergence point between the biosynthesis of C24-desmethyl sterols and cholestenol saponins, and this process can be regulated by MeJA (Ren et al., 2009). We therefore analyzed the levels of compounds related to dioscin biosynthesis in the MeJA treated samples. The level of diosgenin increased after the rhizomes were treated with MeJA, reaching a peak after 12 h and then declining gradually (**Figure 3A**). This suggests that the genes involved in diosgenin biosynthesis were quickly induced in the first few hours, and then, with the increasing expression of glycosyltransferases, diosgenin was converted into dioscin. A total of 47 glycosyltransferase genes were identified in the transcriptome data; as with the differentially expressed genes, the expression pattern of the glycosyltransferases in the aerial and underground portions clustered into different branches. Eight UDP-glycosyltransferase genes (c122117_g2, c118607_g1, c129133_g3, c128767_g2, c129314_g1, c124463_g2, c127876_g2, and c73153_g1) and one glucosaminyltransferase gene (c120589_g1) were highly expressed in rhizomes (**Figure 4A**). Following MeJA treatment, four UDP-glycosyltransferase genes (c122117_g2, c124463_g2, c129314_g1, and c128767_g2) showed positive regulation, while the expression of c73153_g1 and c120598_g1 was suppressed (**Figure 4B**), suggesting the UDP-glycosyltransferase genes (c122117_g2, c118607_g1, c129133_g3, c128767_g2, c129314_g1, c124463_g2, c127876_g2, and c73153_g1) are more likely to be involved in the biosynthesis of dioscin.

**FIGURE 5:**
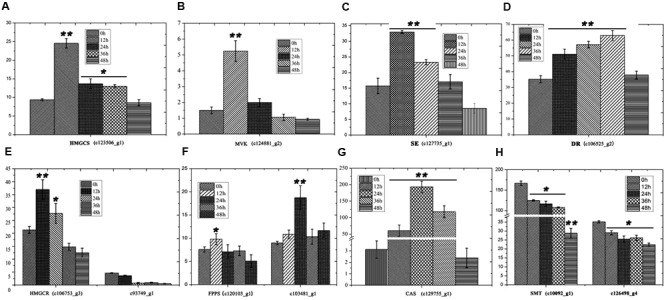
**Expression analysis of genes involved in the biosynthesis of cholesterol. (A)** *HMGS* expression following treatment with MeJA. **(B)** *MVK* expression following treatment with MeJA. **(C)** *SE* expression following treatment with MeJA. **(D)** *DR* expression following treatment with MeJA. **(E)** *HMGCR* expression following treatment with MeJA. **(F)** *FPPS* expression following treatment with MeJA. **(G)** *CAS* expression following treatment with MeJA. Error bars are SD. Statistical significance was determined by Student’s *t*-test (^∗^*p* ≤ 0.01; ^∗∗^*p* ≤ 0.001).

Furthermore, the levels of important precursors of dioscin, such as cholesterol and cycloartenol, were also increased by MeJA treatment, and the level of β-sitosterol, a C-24 methylated metabolic bypass product of cycloartenol, declined (**Figure 3**). This implies that genes involved in the dioscin biosynthesis pathway are up-regulated by MeJA treatment, while those involved in the biosynthesis of C-24 methylated products are suppressed. A total of 137 sequences were annotated as likely taking part in the biosynthesis of dioscin, though only 18 of these were expressed in most of the sampled organs. After treatment with MeJA, these genes exhibited diverse expression patterns. For example, hydroxymethyl glutaric acid CoA synthetase (HMGCS), mevalonate kinase (MVK), hydroxymethyl glutaric acid CoA reductase (HMGCR), squalene epoxidase (SE), 7-dehydrocholesterol reductase (DR), fanesyl pyrophosphate synthase (FPPS), and cycloartenol synthase (CAS) were significantly upregulated (**Figure 5**), suggesting that these genes probably play important roles in the biosynthesis of diosgenin, while the sterol C-24 methyltransferase (SMT) gene, the key gene regulating the carbon flux to cholesterol or C-24 acidylated sterols (Diener et al., 2000; Holmberg et al., 2002), was inhibited by MeJA, in accordance with the decrease in β-sitosterol levels after MeJA treatment.

**FIGURE 6:**
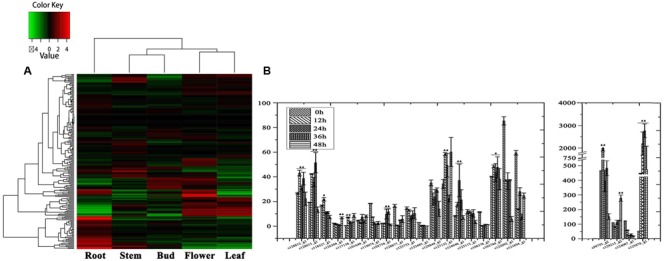
**Expression analysis of the P450 genes. (A)** Organ-specific expression analysis of the P450 genes in *D. nipponica*. **(B)** Expression analysis of the P450 genes highly expressed in roots in MeJA-treated organs. Error bars are SD. Statistical significance was determined by Student’s *t*-test (^∗^*p* ≤ 0.01; ^∗∗^*p* ≤ 0.001).

Cytochrome P450s play major roles in catalyzing the transformation of cholesterol into diosgenin (Dewick, 2011). In the current work, 182 candidate P450s were annotated; these genes had a length more than 300 amino acids and a similarity of over 30% with known P450 genes. Among them, 23 P450 genes belonging to 13 gene subfamilies (CYP71, CYP93, CYP79, CYP81, CYP78, CYP76, CYP704, CYP90, CYP87, CYP722, CYP710, CYP714, and CYP735) were highly expressed in the rhizomes of *D. nipponica* (**Figure 6A**). In addition, 10 candidate P450 sequences, namely CYP93 (c128812_g3), CYP735 (c124940_g1), CYP71 (c128015_g1, c125878_g1, c103784_g1), CYP714 (c117138_g1), CYP78 (c102750_g1), CYP81 (c127132_g1, c129215_g2), and CYP90 (c99793_g2) were found to be up-regulated by MeJA (**Figure 6B**), suggesting that they may be involved in MeJA-related diosgenin biosynthesis. These candidate sequences can be used as bait in further experiments to detect co-expression modules related to diosgenin biosynthesis.

Expression levels of 51 genes putatively encoding transcriptional factors were induced by MeJA treatment (**Supplementary Table S2**). Eight WRKY and eight AP2/ERF family members were found. Jasmonate responsive WRKY and AP2/ERF TF genes were reported in many medicinal plants (Menke et al., 1999; van der Fits and Memelink, 2000; Shoji et al., 2010; Suttipanta et al., 2011). Functional characterizations of them are shown to play important role in controlling specialized pathway of secondary metabolism such as terpene, alkaloid and phenylpropanoid. Further study will focus on elucidating these TF functions for regulating diosgenin and related steroid compounds.

### Weighted Gene Co-expression Network Analysis

**FIGURE 7:**
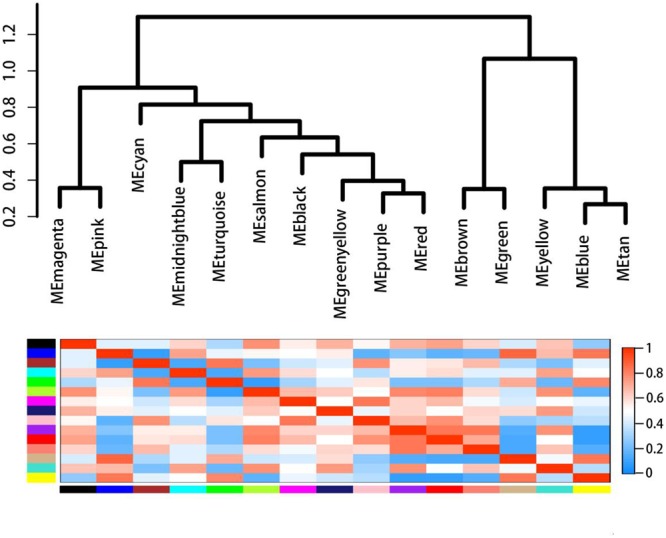
**Gene co-expression modules in MeJA-treated rhizomes showing the cluster dendrogram constructed based on the eigengenes of the modules (above) and the heatmap for the correlation coefficient between the modules (below)**.

In order to further investigate the genes related to the biosynthesis and regulation of dioscin, 28,353 differentially expressed genes in MeJA-treated rhizomes were selected for the construction of a scale-free co-expression network. The weight coefficient parameter β = 9 was chosen to produce a correlation coefficient value of the parameters log (k) and log [p (k)] greater than or equal to 0.8 (**Supplementary Figure S2**). A dynamic hierarchical tree algorithm was used to divide the clustering tree constructed from the differentially expressed genes, resulting in 15 co-expression modules which were named black (545 genes), blue (4,905 genes), brown (4,201 genes), cyan (124 genes), green (2,554 genes), green-yellow (342 genes), magenta (530 genes), yellow (4,070 genes), midnight-blue (29 genes), pink (531 genes), purple (421 genes), red (1,915 genes), salmon (282 genes), tan (321 genes), and turquoise (7,399 genes) (**Figure 7**).

The gene cluster modules were enriched in specific GO functional terms, and statistically significantly enriched terms (*p* ≤ 0.05) were selected for further analysis. The genes in the black module were mainly involved in ribosome biogenesis (*p* = 5.99E-08), carbohydrate metabolic processes (*p* = 0.04), and protein folding (*p* = 0.05); those in the blue module were mainly involved in transcriptional regulation (*p* = 0.008), glyoxylate metabolic processes (*p* = 0.023), sterol biosynthetic processes (*p* = 2.62E-05) and brassinosteroid biosynthetic processes (*p* = 0.034); genes in the brown module were involved in the response to nematodes (*p* = 0.0015), as well as brassinosteroid (*p* = 0.0026) and steroid (*p* = 0.048) metabolic processes; the cyan module was mainly related to the response to fungi (*p* = 0.004) or jasmonic acid (*p* = 0.014), wounding (*p* = 0.013), and jasmonic acid biosynthesis (*p* = 0.006); the genes in the pink module mainly played a role in the biosynthesis of cellulose (*p* = 0.0006) and ethylene (*p* = 0.007) and in the response to ethylene (*p* = 0.01); genes in the purple module were involved in anthocyanin accumulation and in the response to UV light (*p* = 7.36E-05); the genes of the yellow module were involved in the biosynthesis of carotenoids (*p* = 0.001), isopentenyl diphosphate (*p* = 0.02) and terpenoids (*p* = 0.04); and those of the red module were mainly involved in the defense response (*p* = 0.02) and the response to biotic stimulus (*p* = 0.003) and fungi (*p* = 0.03).

These results suggest that the genes in the blue, brown, cyan, red, and yellow modules are probably functionally related to diosgenin biosynthesis. The candidate genes identified were then used as bait to retrieve related genes from these modules and these genes were found to come from seven modules: green–yellow, yellow, blue, brown, turquoise, green, and red. Forty eight genes were retrieved from the green–yellow module; 1,033 genes related to the candidate genes were found in the green module; 540 genes were retrieved from the blue module; 153 genes were retrieved from red module and were annotated to be involved in phenylpropanoid biosynthesis (ko00940); 1591 genes found in the brown module were annotated to play roles in the brassinolide biosynthesis pathway (ko00905); 1,443 genes related to candidate P450s were retrieved from the turquoise module; and 2,381 genes related to glycosyltransferases were retrieved from the yellow module. These data suggest that four co-expression gene modules, namely the blue, brown, yellow, and red modules comprising 4,665 genes, are most likely involved in the MeJA-induced regulation and biosynthesis of dioscin and merit further functional investigation.

## Conclusion

Weighted gene co-expression network analysis of the RNA-Seq data from different organs and MeJA-treated rhizomes of *D. nipponica* identified four gene modules comprising 4,665 genes with properties related to dioscin regulation and biosynthesis. This provides a sound molecular foundation for further characterization of the biosynthesis pathway of dioscin in *D. nipponica* via reverse genetics combined with metabolite profiling. In the future, this will facilitate the enhancement of the production of dioscin, a valuable compound in this important medicinal herb.

## Author Contributions

WS and BW made experiment and finished manuscript. CS and SC were responsible for designing this project as corresponding authors. JY and LL contributed to co-expression analysis. WW and AL contributed to metabolite analysis. LX collected samples.

## Supplementary Material

The Supplementary Material for this article can be found online at: http://journal.frontiersin.org/article/10.3389/fpls.2017.00789/full#supplementary-material

FIGURE S1 **Standard curves for the content determination via LC-MS/MS analysis**.

FIGURE S2 **Parameter selection in the construction of a scale-free co-expression network**.

TABLE S1 **The distribution of percent length coverage for the top matching database entries**.

TABLE S2 **Responsive transcriptional factors induced by MeJA treatment**.

## Conflict of Interest Statement

The authors declare that the research was conducted in the absence of any commercial or financial relationships that could be construed as a potential conflict of interest.

## Footnotes

1 http://transdecoder.github.io/

2 http://wego.genomics.org.cn/cgi-bin/wego/index.pl

3 http://planttfdb.cbi.pku.edu.cn/
