# Supplementary figures and images for: Weighted Gene Co-expression Network Analysis of the Dioscin Rich Medicinal Plant Dioscorea nipponica
Source: Front Plant Sci. 2017 Jun 7;8:789. doi: 10.3389/fpls.2017.00789 (PMC5461258; doi:10.3389/fpls.2017.00789)

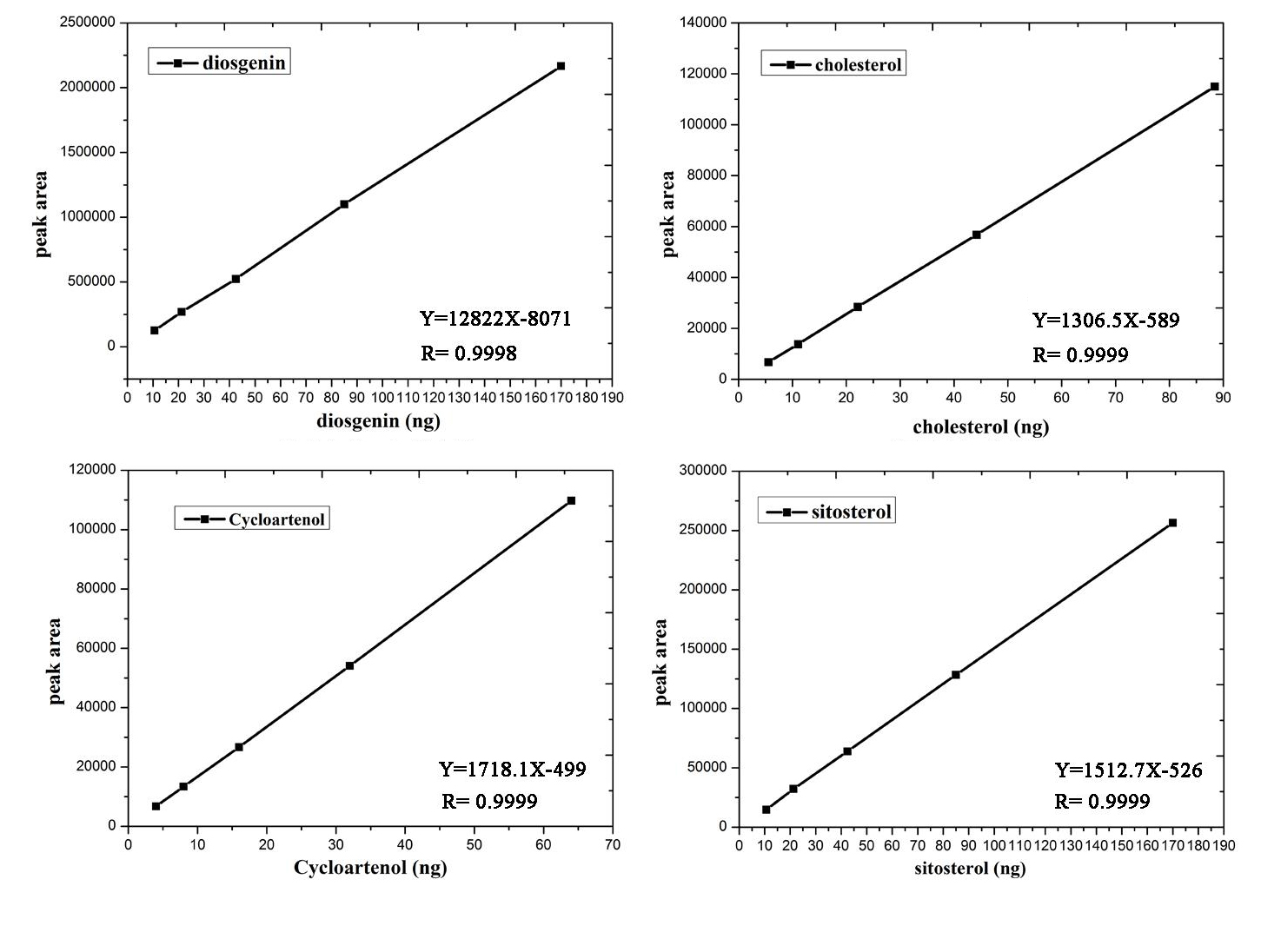

Supplement: FIGURE S1 — Standard curves for the content determination via LC-MS/MS analysis. [file Image_1.JPEG]

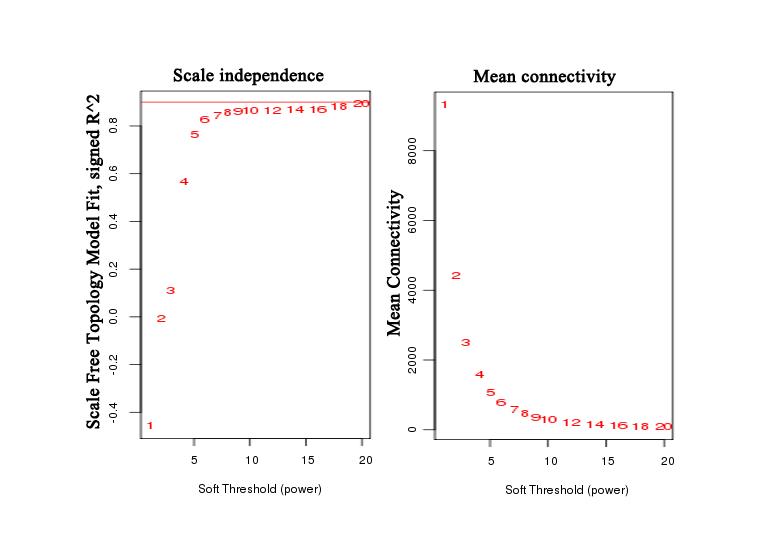

Supplement: FIGURE S2 — Parameter selection in the construction of a scale-free co-expression network. [file Image_2.JPEG]
