# Supplementary material for: Weighted Gene Co-expression Network Analysis of the Dioscin Rich Medicinal Plant Dioscorea nipponica
Source: Front Plant Sci. 2017 Jun 7;8:789. doi: 10.3389/fpls.2017.00789 (PMC5461258; doi:10.3389/fpls.2017.00789)
Supplement: TABLE S1 — The distribution of percent length coverage for the top matching database entries. [file Table_1.DOC]

Table S1 the distribution of percent length coverage for the top matching database entries

| hit_pct_cov_bin^1^ | count_in_bin^2^ | >bin_below^3^ |  | hit_pct_cov_bin | count_in_bin | >bin_below |
| --- | --- | --- | --- | --- | --- | --- |
| 100 | 6,182 | 6,182 |  | 50 | 2,305 | 16,534 |
| 90 | 2,444 | 8,626 |  | 40 | 3,092 | 19,626 |
| 80 | 1,831 | 10,457 |  | 30 | 4,227 | 23,853 |
| 70 | 1,783 | 12,240 |  | 20 | 5,168 | 29,021 |
| 60 | 1,989 | 14,229 |  | 10 | 2,268 | 31,289 |

^1^ The percentage. ^2^ There are X proteins that each match a Trinity transcript by >n % and ≦ (n+10) % of their protein lengths. ^3^There are X proteins that are represented by nearly full-length transcripts, having >n % alignment coverage.
